# Supplementary material for: Alzheimer’s disease plasma biomarkers are associated with cognitive performance among Hispanic/Latino adults
Source: Commun Med (Lond). 2025 Dec 15;6:33. doi: 10.1038/s43856-025-01295-7 (PMC12815935; doi:10.1038/s43856-025-01295-7)
Supplement: Supplementary file 2 — Supplementary Materials [file 43856_2025_1295_MOESM2_ESM.pdf]

Supplement

Tables

Supplementary Table 1. Means and standard deviations of plasma biomarkers and cognitive performance in the SOL-INCA target population overall and by age group.

	Overall (N=5730)	<60 Years (N=2416)	60-69 Years (N=2346)	70+ Years (N=968)	
	Mean (SD)	Mean (SD)	Mean (SD)	Mean (SD)	P-value
**Aβ40**	117.6 (27.5)	109.1 (21.7)	116.5 (27.2)	132.1 (26.5)	2.21×10^{-39}
**Aβ42**	8.1 (2.1)	7.7 (1.9)	8.0 (2.2)	8.8 (2.0)	1.12×10^{-18}
**Aβ42/40**	0.1 (0.0)	0.1 (0.0)	0.1 (0.0)	0.1 (0.0)	5.06×10^{-08}
**pTau-181**	2.0 (1.7)	1.7 (2.0)	2.0 (1.6)	2.6 (1.4)	8.27×10^{-22}
**NfL**	20.2 (21.5)	14.4 (14.1)	19.8 (24.3)	29.1 (21.0)	6.70×10^{-29}
**GFAP**	159.4 (85.7)	119.9 (60.4)	155.0 (77.5)	223.3 (79.7)	5.45×10^{-99}
**ln(Aβ40)**	4.7 (0.2)	4.7 (0.2)	4.7 (0.2)	4.9 (0.2)	2.26×10^{-40}
**ln(Aβ42)**	2.1 (0.3)	2.0 (0.2)	2.1 (0.3)	2.1 (0.2)	1.10×10^{-17}
**ln(Aβ42/40)**	-2.7 (0.2)	-2.7 (0.2)	-2.7 (0.2)	-2.7 (0.2)	1.11×10^{-06}
**ln(pTau-181)**	0.6 (0.5)	0.4 (0.5)	0.6 (0.5)	0.8 (0.4)	9.19×10^{-46}
**ln(NfL)**	2.8 (0.6)	2.5 (0.5)	2.8 (0.5)	3.2 (0.4)	8.00×10^{-107}
**ln(GFAP)**	5.0 (0.5)	4.7 (0.4)	5.0 (0.5)	5.3 (0.3)	2.70×10^{-116}
**B-SEVLT-Sum (raw score)**	22.5 (5.9)	24.3 (5.8)	22.6 (6.0)	19.5 (4.7)	1.67×10^{-40}
**B-SEVLT-Recall (raw score)**	8.1 (3.0)	9.0 (3.0)	8.2 (3.1)	6.7 (2.3)	8.32×10^{-41}
**Word Fluency (raw score)**	18.0 (7.4)	19.2 (7.6)	17.8 (7.7)	16.5 (6.1)	6.25×10^{-10}
**Digit Symbol Substitution (raw score)**	31.5 (13.3)	37.1 (13.3)	31.1 (12.6)	23.6 (9.8)	4.45×10^{-62}
**Global cognition (average of z-scores)**	-0.0 (0.8)	0.3 (0.8)	-0.0 (0.8)	-0.4 (0.6)	4.94×10^{-55}

Note 1: Results are derived using data from SOL-INCA (unweighted n = 5,730) using survey-weighted means and proportions, with differences across age groups assessed using adjusted F-tests and chi-square tests (two-sided tests).
Note 2: All reported values are weighted to represent the SOL-INCA target population.
Note 3: No multiple comparison adjustment is applied given the descriptive purpose of the table.

Abbreviations: Aβ = amyloid-beta, B-SEVLT = Brief-Spanish English Verbal Learning Test, GFAP = glial fibrillary acidic protein, NfL= neurofilament light, pTau = phosphorylated tau, SD = standard deviation

Supplementary Table 2. Benjamini-Hochberg false discovery rate correction for multiple comparisons per outcome.

	b [95% CIs]	Uncorrected *p*-value	BH-adjusted *p*-value
	**Global Cognition**		
ln(Aβ42/40)	0.09 [-0.03;0.21]	0.138	0.184
ln(pTau-181)	-0.06 [-0.12;-0.01]	0.022	0.045
ln(NfL)	-0.07 [-0.12;-0.02]	0.005	0.019
ln(GFAP)	-0.03 [-0.1;0.04]	0.362	0.362
	**B-SEVLT-Sum**		
ln(Aβ42/40)	0.12 [-0.04;0.28]	0.142	0.142
ln(pTau-181)	-0.09 [-0.16;-0.01]	0.021	0.058
ln(NfL)	-0.08 [-0.15;-0.01]	0.029	0.058
ln(GFAP)	-0.09 [-0.18;0.01]	0.074	0.099
	**B-SEVLT-Recall**		
ln(Aβ42/40)	0.00 [-0.18;0.18]	0.986	0.986
ln(pTau-181)	-0.09 [-0.17;-0.01]	0.021	0.085
ln(NfL)	-0.04 [-0.11;0.04]	0.299	0.399
ln(GFAP)	-0.05 [-0.14;0.04]	0.241	0.399
	**Word Fluency**		
ln(Aβ42/40)	0.18 [0.01;0.34]	0.041	0.131
ln(pTau-181)	-0.02 [-0.11;0.06]	0.545	0.584
ln(NfL)	-0.07 [-0.13;0]	0.066	0.131
ln(GFAP)	0.02 [-0.06;0.11]	0.584	0.584
	**Digit Symbol Substitution**		
ln(Aβ42/40)	0.07 [-0.07;0.21]	0.344	0.459
ln(pTau-181)	-0.06 [-0.13;0.01]	0.091	0.182
ln(NfL)	-0.11 [-0.17;-0.05]	<0.001	0.001
ln(GFAP)	0.00 [-0.07;0.07]	0.975	0.975

Note 1: Results are derived from survey-weighted linear regression models (two-sided tests) using data from SOL-INCA (unweighted n = 5,730).

Note 2: Global cognition score is average of z-scored domain-specific cognitive test scores (M[SD]=-0.00[0.79]).

Note 3: Results are from the full covariate models adjusted for age, sex, education, Hispanic/Latino background, field center, BMI, diabetes, hypertension, dyslipidemia, CKD, and APOE ε4 genotype.
Note 4: Values near zero may appear as ±0.00.
Note 5: False discovery rate (Benjamini-Hochberg) was applied within each outcome.
Abbreviations: Aβ = amyloid-beta, b = regression coefficient, BH = Benjamini-Hochberg, B-SEVLT = Brief-Spanish English Verbal Learning Test, CI = confidence interval, GFAP = glial fibrillary acidic protein, NfL= neurofilament light, pTau = phosphorylated tau

Supplementary Table 3. Associations between NfL and cognitive performance using the multiplex assay

	M0	M1	M2	M3	M4	M5
	**b[CI95]**	**b[CI95]**	**b[CI95]**	**b[CI95]**	**b[CI95]**	**b[CI95]**
			**Global Cognition**			
ln(NfL)	-0.35 [-0.41;-0.28]	-0.10 [-0.16;-0.04]	-0.10 [-0.15;-0.05]	-0.09 [-0.14;-0.04]	-0.07 [-0.12;-0.02]	-0.07 [-0.12;-0.02]
p-value	5.46×10^{-23}	0.002	1.29×10^{-04}	0.001	0.010	0.010
			**B-SEVLT-Sum**			
ln(NfL)	-0.40 [-0.48;-0.32]	-0.14 [-0.22;-0.07]	-0.11 [-0.18;-0.04]	-0.10 [-0.17;-0.03]	-0.08 [-0.15;-0.00]	-0.07 [-0.15;-0.00]
p-value	9.53×10^{-21}	2.82×10^{-04}	0.002	0.006	0.042	0.044
			**B-SEVLT-Recall**			
ln(NfL)	-0.36 [-0.44;-0.28]	-0.10 [-0.18;-0.02]	-0.07 [-0.14;-0.00]	-0.05 [-0.12;0.02]	-0.03 [-0.10;0.05]	-0.02 [-0.10;0.05]
p-value	1.15×10^{-17}	0.013	0.042	0.155	0.510	0.532
			**Word Fluency**			
ln(NfL)	-0.18 [-0.26;-0.11]	-0.06 [-0.14;0.01]	-0.08 [-0.15;-0.01]	-0.08 [-0.15;-0.01]	-0.07 [-0.14;0.00]	-0.07 [-0.14;0.00]
p-value	9.22×10^{-07}	0.081	0.022	0.024	0.065	0.053
			**Digit Symbol Substitution**			
ln(NfL)	-0.44 [-0.52;-0.36]	-0.09 [-0.17;-0.01]	-0.13 [-0.20;-0.07]	-0.12 [-0.18;-0.06]	-0.11 [-0.16;-0.05]	-0.11 [-0.16;-0.05]
p-value	1.23×10^{-23}	0.022	1.78×10^{-05}	9.16×10^{-05}	4.49×10^{-04}	4.06×10^{-04}

Note 1: Results are derived from survey-weighted linear regression models (two-sided tests) using data from SOL-INCA, limited to the sample with NfL assayed using multiple assay (n = 5,562).
Note 2: Global cognition score is average of z-scored domain-specific cognitive test scores (M[SD]=-0.00[0.79]).
Note 3: M0 is an unadjusted model; M1 is adjusted for age; M2 is additionally adjusted for sex, education, Hispanic/Latino background, and field center; M3 is additionally adjusted for BMI, diabetes, hypertension, and dyslipidemia; M4 is additionally adjusted for CKD; and M5 is additionally adjusted for APOE ε4 genotype.
Note 4: Values near zero may appear as ±0.00.
Note 5: No multiple comparison adjustment is applied.
Abbreviations: b = regression coefficient, B-SEVLT = Brief-Spanish English Verbal Learning Test, CI = confidence interval, NfL= neurofilament light

Supplementary Figure 1. Flowchart showing participant inclusion and exclusion.

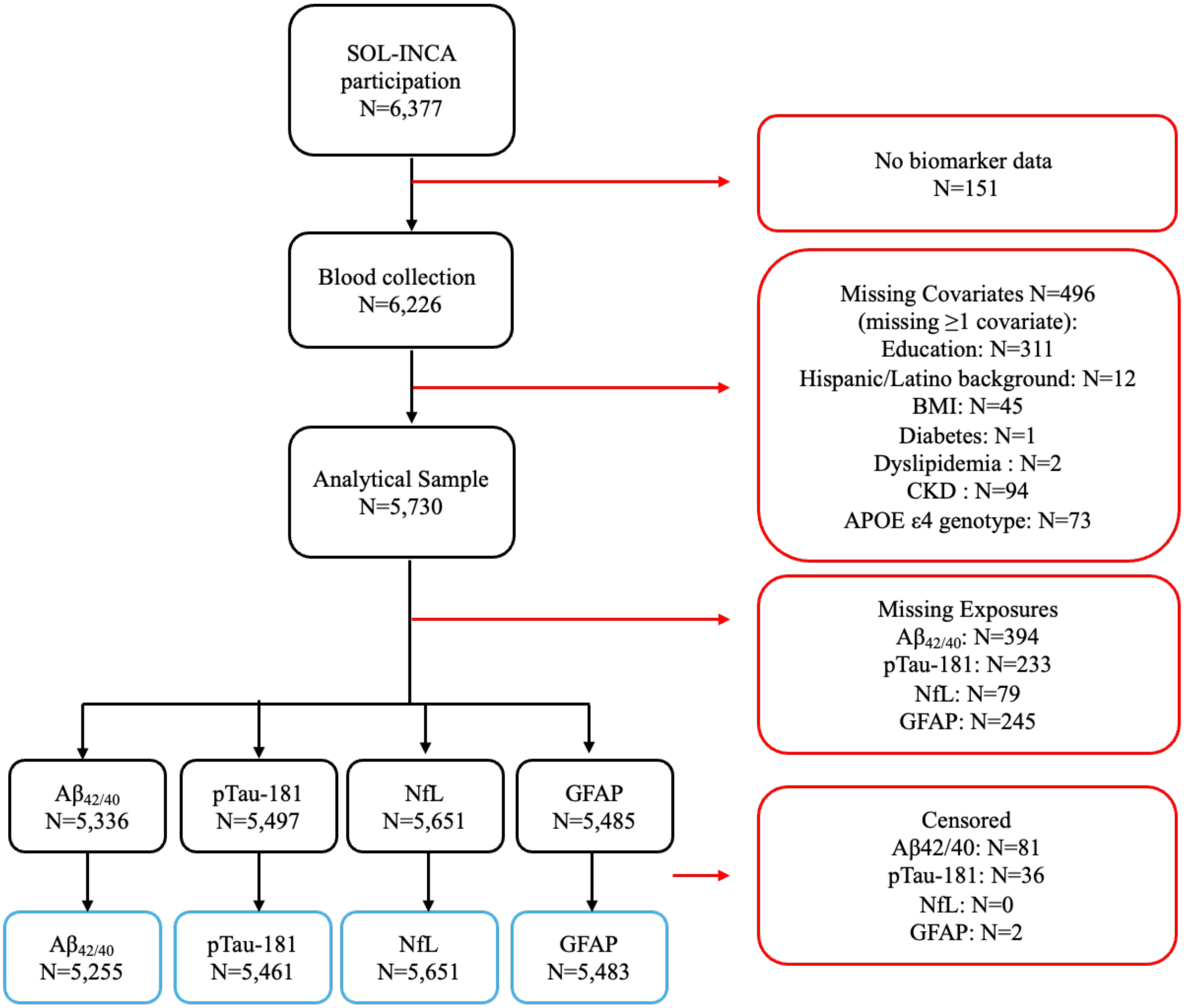

Abbreviations: Aβ = amyloid-beta, GFAP = glial fibrillary acidic protein, NfL= neurofilament light, pTau = phosphorylated tau, SOL-INCA = Study of Latinos-Investigation of Neurocognitive Aging. Sample sizes for individual outcomes varied slightly due to missing cognitive test scores between unweighted N=5,655 for Digit Symbol Substitution and unweighted N=5,711 for B-SEVLT-Sum

Supplementary Figure 2. Correlation matrix of log-transformed plasma biomarkers and cognitive performance in the SOL-INCA target population

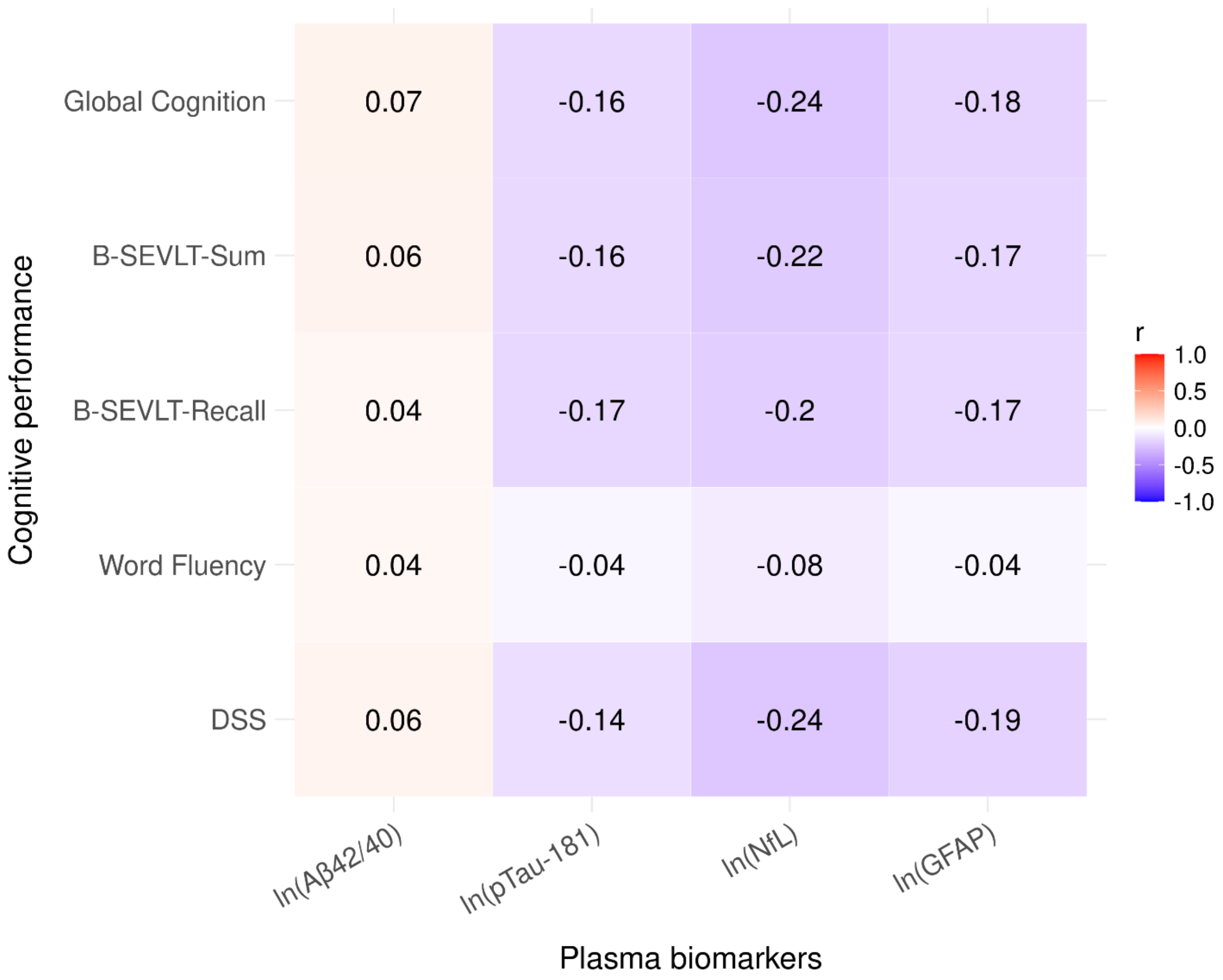

Note 1. Results are derived from survey weighted Pearson's correlations using data from SOL-INCA (unweighted n = 5,730).

Abbreviations. Aβ = amyloid-beta, B-SEVLT = Brief-Spanish English Verbal Learning Test, GFAP = glial fibrillary acidic protein, NfL= neurofilament light, pTau = phosphorylated tau

Supplementary Figure 3. Scatter plots of log-transformed plasma biomarkers and cognitive performance with linear regression line in the SOL-INCA target population

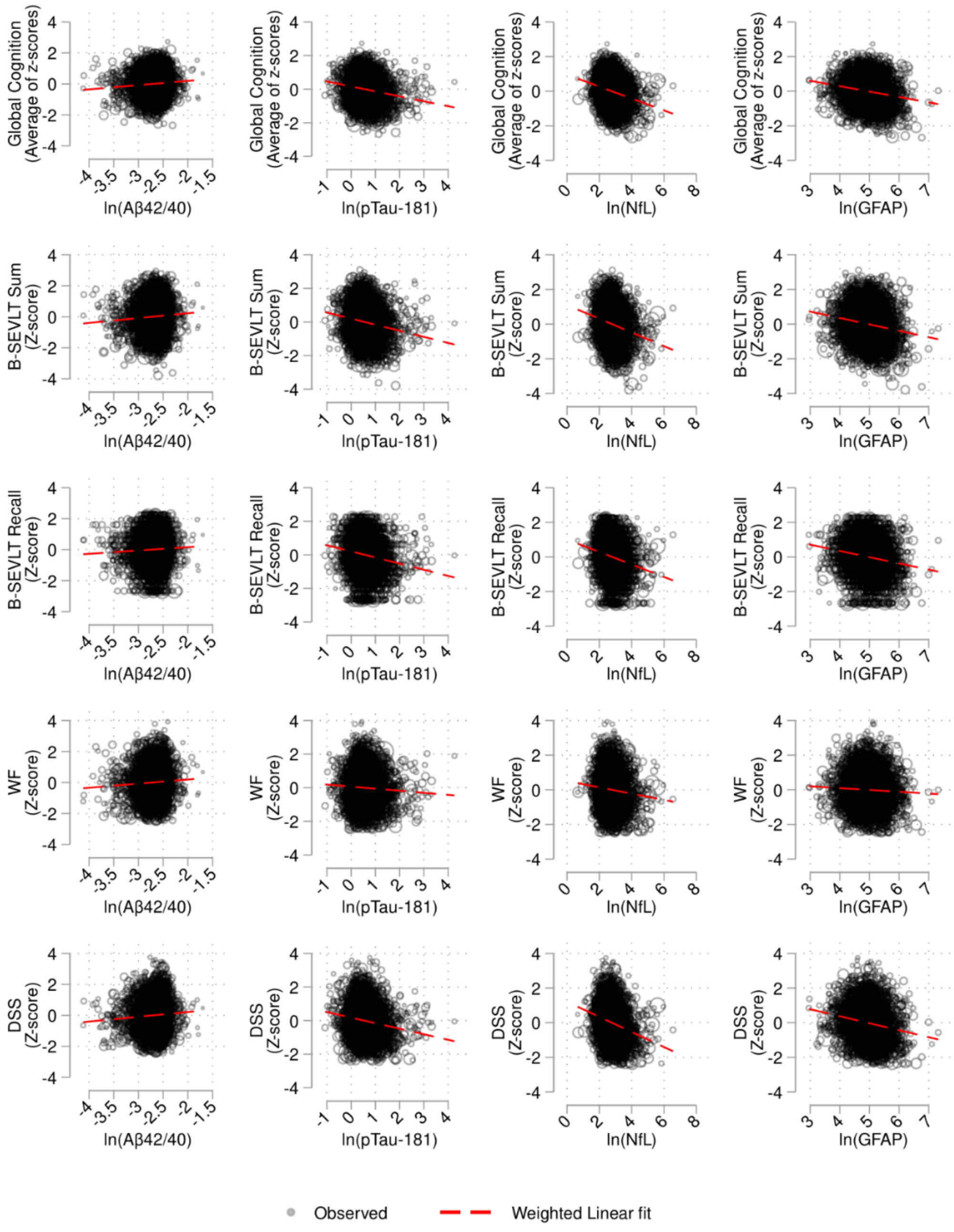

Note 1. The scatter plot and linear regression line are weighted to represent the target population of SOL-INCA (unweighted n = 5,730).
Abbreviations. Aβ = amyloid-beta, B-SEVLT = Brief-Spanish English Verbal Learning Test, GFAP = glial fibrillary acidic protein, NfL= neurofilament light, pTau = phosphorylated tau

Supplementary Figure 4. Scatter plots of log-transformed plasma biomarkers and cognitive performance with linear regression line in the SOL-INCA target population, by age group

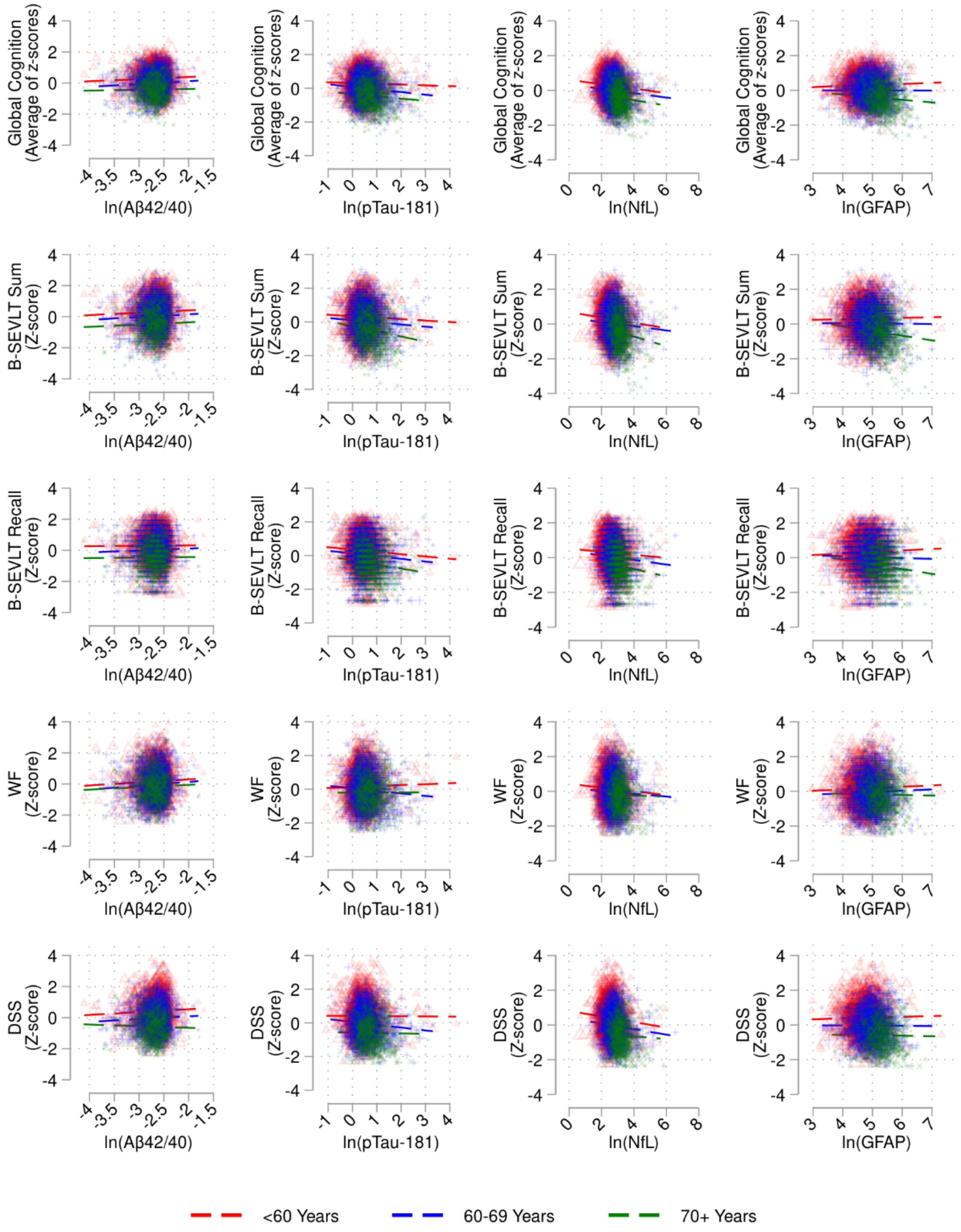

Note 1. The scatter plot and linear regression line are weighted to represent the target population of SOL-INCA (unweighted n = 5,730).

Abbreviations. Aβ = amyloid-beta, B-SEVLT = Brief-Spanish English Verbal Learning Test, GFAP = glial fibrillary acidic protein, NfL= neurofilament light, pTau = phosphorylated tau

Supplementary Figure 5. Scatter plots of log-transformed plasma biomarkers and cognitive performance with linear regression line in the SOL-INCA target population, by cognitive status

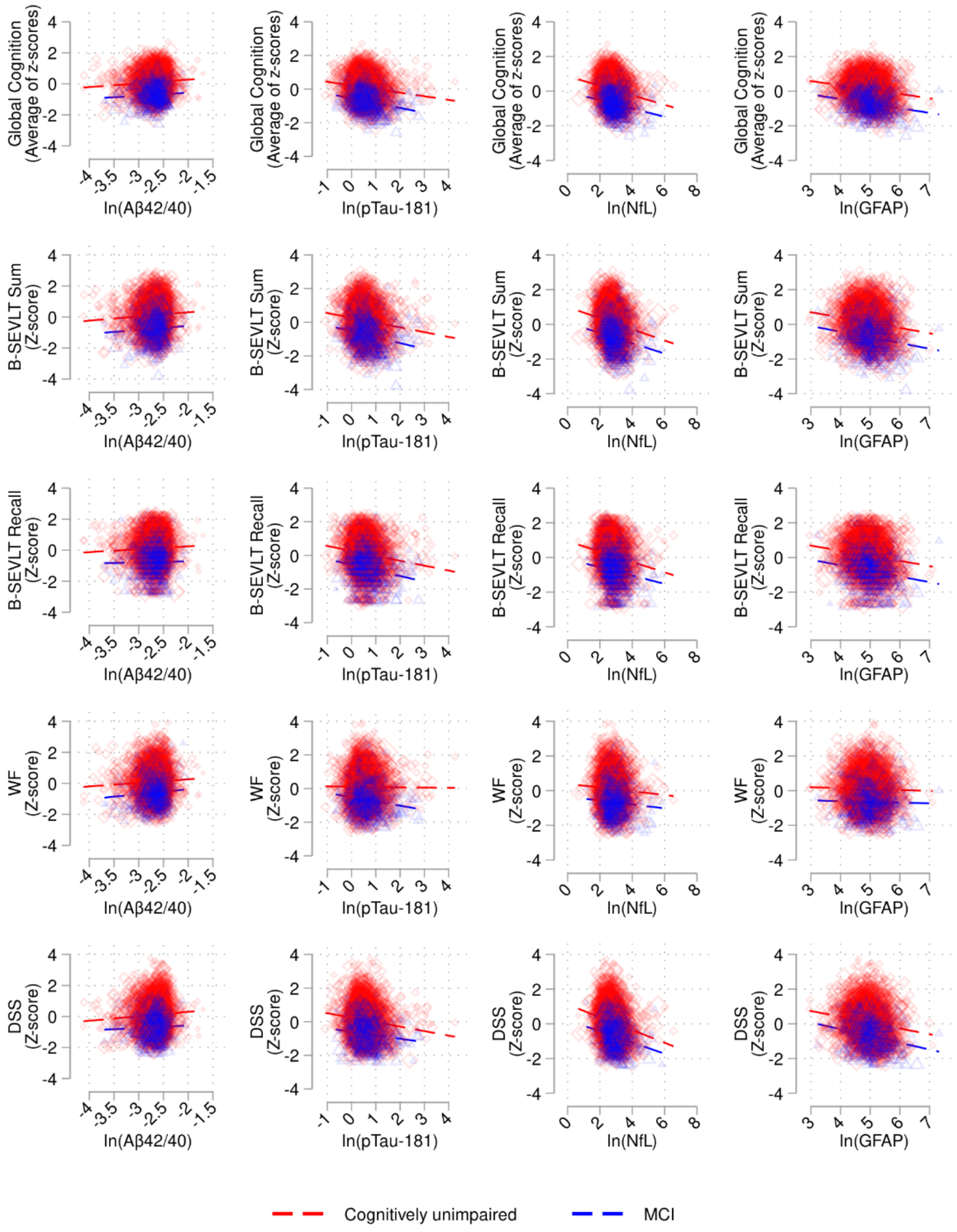

Note 1. The scatter plot and linear regression line are weighted to represent the target population of SOL-INCA (unweighted n = 5,730).

Abbreviations. Aβ = amyloid-beta, B-SEVLT = Brief-Spanish English Verbal Learning Test, GFAP = glial fibrillary acidic protein, MCI = mild cognitive impairment, NfL= neurofilament light, pTau = phosphorylated tau

Supplementary Figure 6. Associations between log-transformed plasma biomarkers and global cognitive performance in the SOL-INCA target population, with additional models including all log-transformed biomarkers simultaneously.

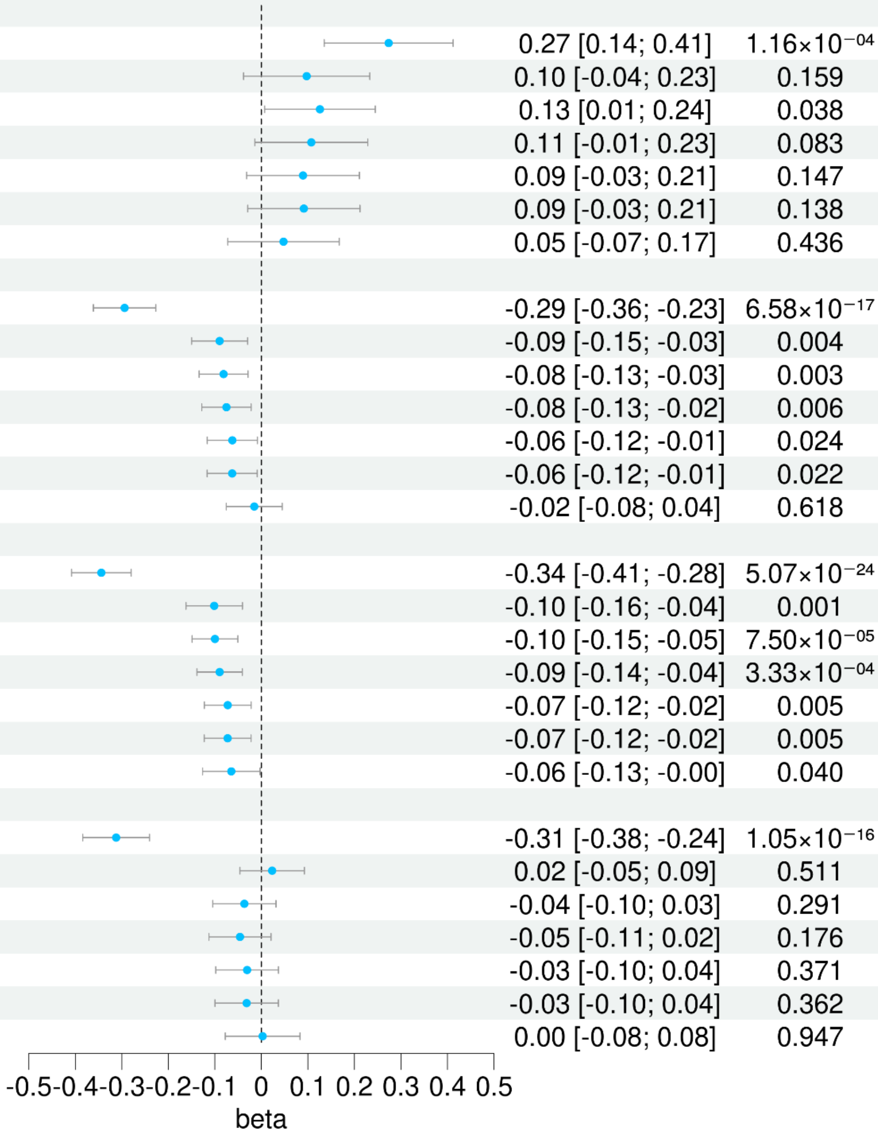

Global Cognition	Estimate [95% CI]	P
**ln(Aβ42/40)**		
M0	0.27 [0.14; 0.41]	1.16×10^{-04}
M1	0.10 [-0.04; 0.23]	0.159
M2	0.13 [0.01; 0.24]	0.038
M3	0.11 [-0.01; 0.23]	0.083
M4	0.09 [-0.03; 0.21]	0.147
M5	0.09 [-0.03; 0.21]	0.138
M6	0.05 [-0.07; 0.17]	0.436
**ln(pTau-181)**		
M0	-0.29 [-0.36; -0.23]	6.58×10^{-17}
M1	-0.09 [-0.15; -0.03]	0.004
M2	-0.08 [-0.13; -0.03]	0.003
M3	-0.08 [-0.13; -0.02]	0.006
M4	-0.06 [-0.12; -0.01]	0.024
M5	-0.06 [-0.12; -0.01]	0.022
M6	-0.02 [-0.08; 0.04]	0.618
**ln(NfL)**		
M0	-0.34 [-0.41; -0.28]	5.07×10^{-24}
M1	-0.10 [-0.16; -0.04]	0.001
M2	-0.10 [-0.15; -0.05]	7.50×10^{-05}
M3	-0.09 [-0.14; -0.04]	3.33×10^{-04}
M4	-0.07 [-0.12; -0.02]	0.005
M5	-0.07 [-0.12; -0.02]	0.005
M6	-0.06 [-0.13; -0.00]	0.040
**ln(GFAP)**		
M0	-0.31 [-0.38; -0.24]	1.05×10^{-16}
M1	0.02 [-0.05; 0.09]	0.511
M2	-0.04 [-0.10; 0.03]	0.291
M3	-0.05 [-0.11; 0.02]	0.176
M4	-0.03 [-0.10; 0.04]	0.371
M5	-0.03 [-0.10; 0.04]	0.362
M6	0.00 [-0.08; 0.08]	0.947

Note 1: Results are derived from survey-weighted linear regression models (two-sided tests) using data from SOL-INCA (unweighted n = 5,730).
Note 2: Global cognition score is average of z-scored domain-specific cognitive test scores (M[SD]=-0.00[0.79]).
Note 3: M0 is an unadjusted model; M1 is adjusted for age; M2 is additionally adjusted for sex, education, Hispanic/Latino background, and field center; M3 is additionally adjusted for BMI, diabetes, hypertension, and dyslipidemia; M4 is additionally adjusted for CKD; M5 is additionally adjusted for APOE ε4 genotype; and M6 includes all log-transformed plasma biomarkers simultaneously and is adjusted for all covariates.

Note 4: Points depict survey-weighted adjusted marginal means and error bars depict 95% confidence intervals.
Note 5: Values near zero may appear as ±0.00.
Note 6: No multiple comparison adjustment is applied.

Abbreviations: Aβ = amyloid-beta, b = regression coefficient, B-SEVLT = Brief-Spanish English Verbal Learning Test, CI = confidence interval, DSS = Digit Symbol Substitution, GFAP = glial fibrillary acidic protein, NfL= neurofilament light, pTau = phosphorylated tau

Supplementary Figure 7. Associations between log-transformed plasma biomarkers and domain-specific cognitive performance in the SOL-INCA target population, with additional models including all log-transformed biomarkers simultaneously.

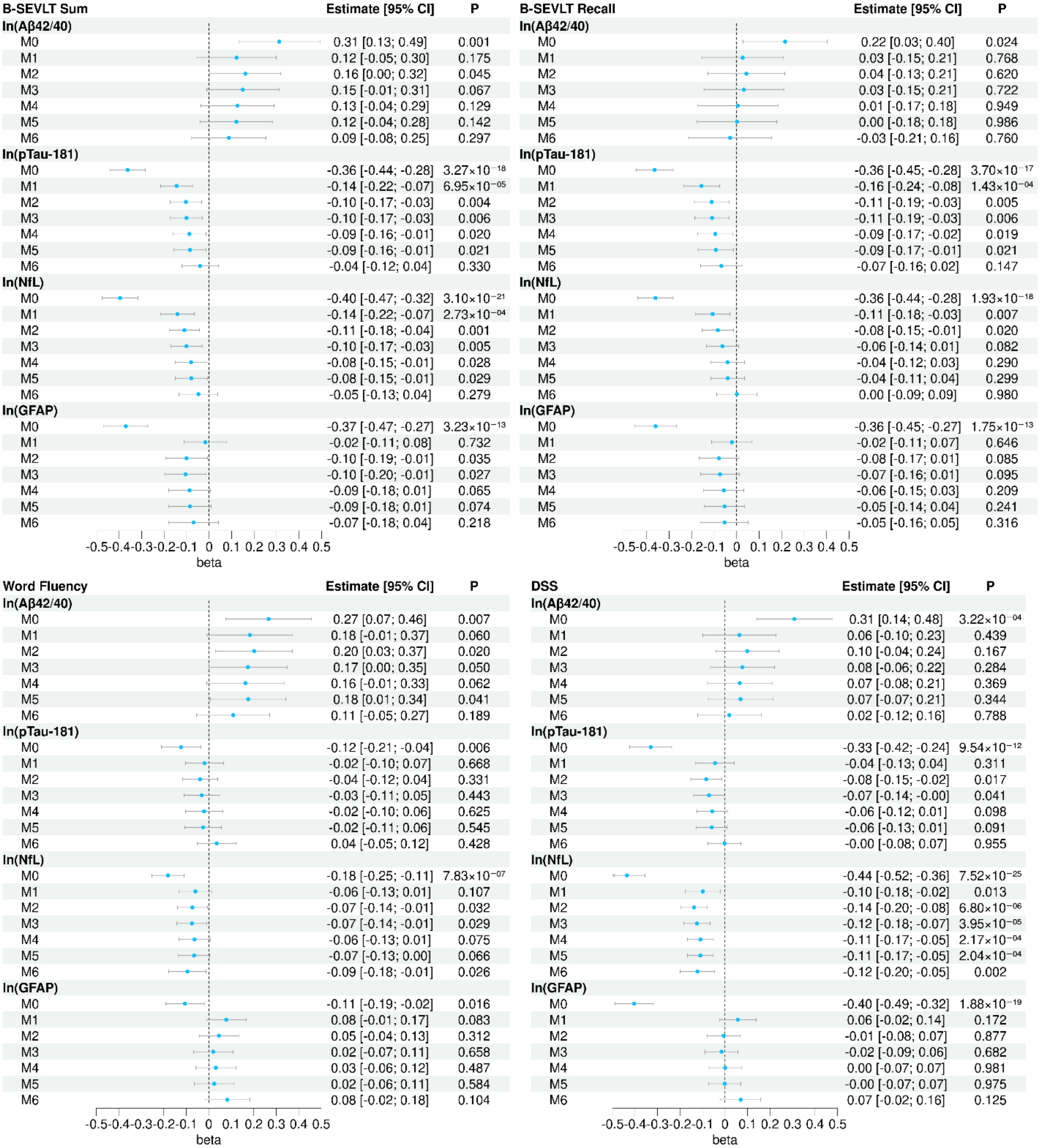

Note 1: Results are derived from survey-weighted linear regression models (two-sided tests) using data from SOL-INCA (unweighted n = 5,730).
Note 2: Global cognition score is average of z-scored domain-specific cognitive test scores (M[SD]=-0.00[0.79]).
Note 3: M0 is an unadjusted model; M1 is adjusted for age; M2 is additionally adjusted for sex, education, Hispanic/Latino background, and field center; M3 is additionally adjusted for BMI,

diabetes, hypertension, and dyslipidemia; M4 is additionally adjusted for CKD; M5 is additionally adjusted for APOE ε4 genotype; and M6 includes all log-transformed plasma biomarkers simultaneously and is adjusted for all covariates
Note 4: Points depict survey-weighted adjusted marginal means and error bars depict 95% confidence intervals.
Note 5: Values near zero may appear as ±0.00.
Note 6: No multiple comparison adjustment is applied.

Abbreviations: Aβ = amyloid-beta, b = regression coefficient, B-SEVLT = Brief-Spanish English Verbal Learning Test, CI = confidence interval, DSS = Digit Symbol Substitution, GFAP = glial fibrillary acidic protein, NfL= neurofilament light, pTau = phosphorylated tau
