# Supplementary material for: Alzheimer’s disease plasma biomarkers are associated with cognitive performance among Hispanic/Latino adults
Source: Commun Med (Lond). 2025 Dec 15;6:33. doi: 10.1038/s43856-025-01295-7 (PMC12815935; doi:10.1038/s43856-025-01295-7)
Supplement: Supplementary file 3 — Description of Additional Supplementary files [file 43856_2025_1295_MOESM3_ESM.docx]

**Description of Additional Supplementary Files**

Supplementary Data 1: Associations between demographic and cog

Supplementary Data 2: main associations table

Supplementary Data 3: Agestratified association

Supplementary Data 4: Descriptive by cognitive status

Supplementary Data 5: Cogstatus-stratified association

Supplementary Data 6: allbiomarker associations table

Supplementary Data 7: Associations between ATN and cog - exclude 3SD outliers
